# Endogenous Therapeutic Agents for Acute Corneal and Ocular Surface Injuries

**DOI:** 10.1167/tvst.15.3.16

**Published:** 2026-03-18

**Authors:** Christian Reinhardt, Joshua Ong, Kyung-No Son, Vinay Kumar Aakalu

**Affiliations:** 1 Department of Ophthalmology and Visual Sciences, University of Michigan Kellogg Eye Center, Ann Arbor, Michigan, USA

**Keywords:** endogenous therapeutics, acute corneal injury, cornea, ocular surface, external disease

## Abstract

Acute corneal injuries vary greatly in morbidity, but can cause severe cases of vision loss. Among these injuries are mechanical corneal traumas and acute chemical burns. The current standard of care in the acute phase of management is aimed at reducing the offending agent, pain management, and infection prevention; however, corneal scarring and limbal stem cell deficiency may result even after the acute management of severe corneal injuries. In the most serious cases, surgical intervention, including cornea or stem cell transplantation, may be required to restore functional vision. As such, there is a great need for additional therapies in the acute phase of management that can help to reduce long-term, vision-reducing outcomes. Certain endogenous agents have emerged as a therapeutic class that may be used for the promotion of corneal wound healing with the goal of reducing the odds of requiring invasive surgery. In this review, we provide an overview of novel endogenous therapeutic agents for the treatment of acute corneal trauma and acute chemical burns. The endogenous therapeutic agents explored include amnion, exosomes, stem cells, platelet-rich plasma, peptides, growth factors, and umbilical cord serum. The results of these studies indicate encouraging potential for endogenous agents to facilitate corneal recovery and vision preservation. However, because many of these agents are in the early stages of development and clinical trials, further research is required to understand their efficacy in the clinical setting.

## Introduction

Acute corneal injuries vary greatly in morbidity ranging from minor injury with full recovery to blinding injuries requiring invasive interventions or even enucleation.^1^ Acute corneal injuries include corneal abrasions, perforations, chemical burns, and microbial keratitis. Treatments of these injuries involve the removal of foreign bodies, antibiotics, and neutralization of acids/alkalis, followed by management of pain and infection prevention.^1^ However, corneal scarring and recurrent corneal erosion have been noted among the limitations in the treatment of corneal injuries.^1^ Significant central corneal scarring may require a corneal transplant surgery; thus, efforts are focused on improving the healing process to reduce the need for an invasive surgery.

Physiologically, the healing process of the corneal epithelium involves proliferation, migration, and differentiation to reform the corneal epithelium.^2^ These processes may be further optimized with the emergence of endogenous therapeutic agents. These novel agents are often rich in therapeutic properties, which may allow for decreased inflammation and improvement in corneal recovery. Supplementation with current clinical standard protocols for corneal injuries may help to reduce corneal scarring and help preserve vision. Although in an area of therapeutic promise, many of these agents are still early in development and are not yet in full clinical adoption. In this article, we discuss in depth the status and future directions of endogenous therapeutic agents for acute corneal injuries, including corneal trauma and chemical burns. The endogenous therapeutic agents described in this paper include amniotic membrane (AM), umbilical cord blood serum (UCBS), platelet-rich plasma (PRP), exosomes, peptides, and stem cells.

## Acute Corneal Injuries and Endogenous Treatments

We discuss acute corneal injuries and their epidemiology and pathophysiology. We then discuss current research in endogenous therapeutics for these. We cover primarily acute injuries including acute corneal trauma and chemical burns. Although other pathologies may benefit from endogenous therapeutic agents and have been studied in this setting, the inclusion of these nonacute pathologies are outside of the scope of this article.

### Acute Corneal Trauma

Corneal trauma refers to anything that injures the cornea. Corneal trauma includes corneal abrasions and perforations. Foreign bodies, such as contact lens or fingernails, can cause corneal abrasions. Approximately 43 million people are blind worldwide and more than 55 million eye injuries are reported globally, making ocular trauma the leading cause of unilateral blindness.^3^ These injuries account for 80% of all eye trauma cases.^1^^,^^4^^,^^5^ One retrospective study showed that injuries to the anterior segment among pediatric eye injuries are the most common, and approximately 1 in 20 ocular injuries required surgical intervention; 1 in 13 had permanently decreased vision.^6^ More severely, corneal perforation is caused by higher-speed projectiles/foreign bodies entering the eye, subsequently lacerating or perforating it and damaging the corneal epithelium at the minimum.^1^ Low- and middle-income countries are disproportionately affected by ocular trauma^3^

**Figure 1.**
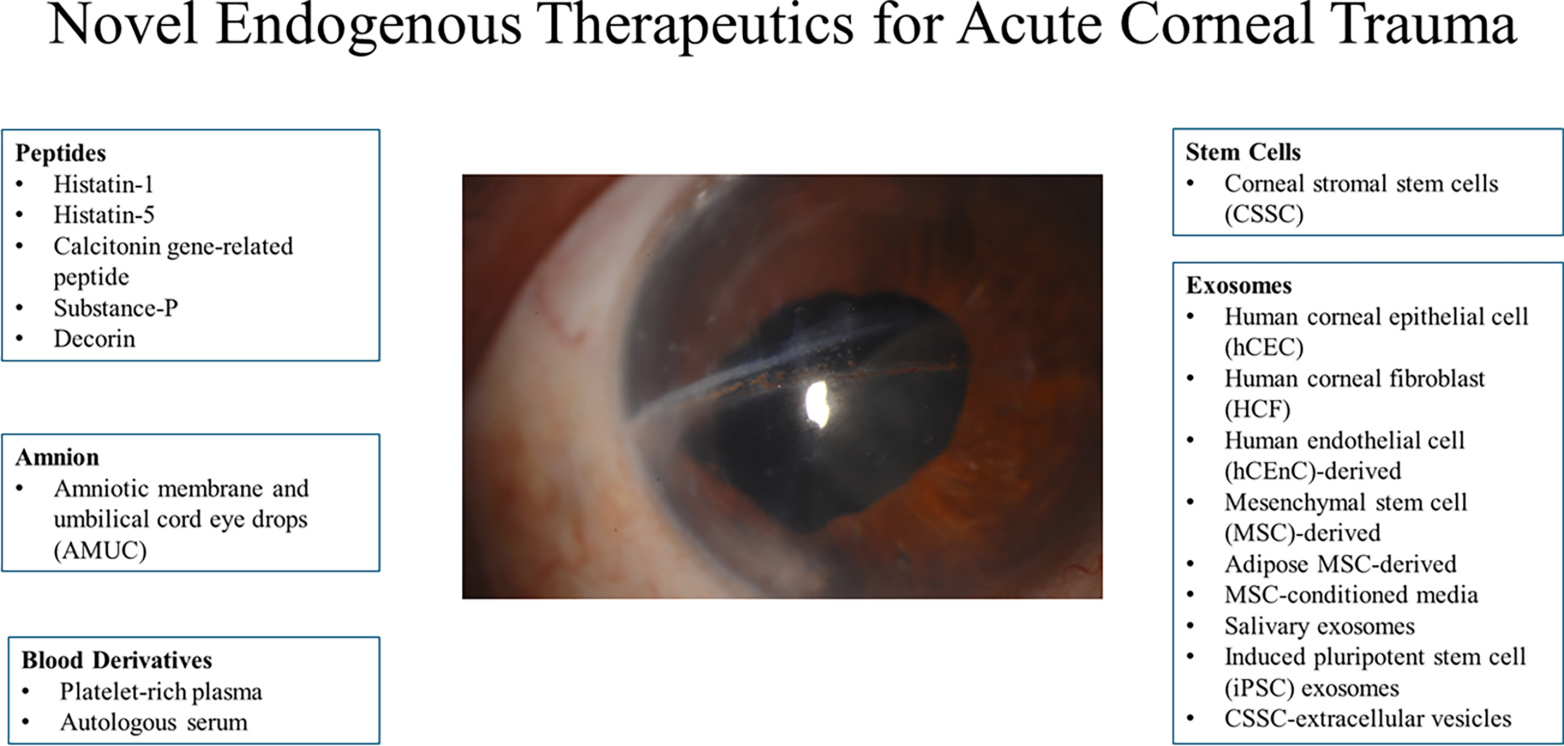
Summary of therapeutics for acute corneal trauma. Central image shows corneal horizontal corneal scarring. (Central image adapted from Awidi et al.^8^ Corneal lacerations following crab claw injuries. Am J Ophthalmol Case Rep. 2022 Jan 20;25:101288. with permission under Creative Commons License.)

These injuries are managed by minimizing pain and preventing infections while maximizing corneal wound healing. Standard management consists of oral analgesics (i.e. narcotics, acetaminophen, nonsteroidal anti-inflammatory drugs) or topical anesthetics (proparacaine hydrochloride, and tetracaine hydrochloride) for pain, prophylactic topical antibiotics for infection prevention, lubricant drops as needed, and daily symptom monitoring.^7^ However, standard management does not use treatments that are directed toward the acceleration of corneal wound healing. Here, we review amnion, exosomes, stem cells, PRP, and peptides as endogenous/allogenic therapies for corneal trauma. Figure 1 summarizes these therapies. It is important to note that therapeutics such as AM and UCBS are not truly endogenous, but biologically derived/originating from allogenic tissues. However, this research is highly relevant and therefore included within the discussion of this review.

**Table 1.** Exosome Treatment in Acute Corneal Trauma.

Exosome Source	Level of Evidence	Key Outcomes	Clinical Relevance	Reference
hCEC-, hCF-, and hCEnC-derived exosomes	In vitro (scratch-wounded hCECs)	All exosome groups accelerated wound closure vs. control; hCEC-exo produced fastest closure; hCF-exo most increased proliferation; signaling differences (↑HSP27 with hCEC-exo; ↑p-GSK-3β/↑p38α/↑β-catenin with hCEC- and hCF-exo; these phosphorylation effects abolished by hCEnC-exo)	Supports cornea-derived exosomes as prohealing agents and shows cell-type–specific effects relevant to therapeutic selection/optimization	18
MSC-exo	In vitro + in vivo (rat irrPTK injury model)	Faster closure; increased proliferation/migration (scratch assay); improved re-epithelialization by day 5 in vivo; reduced stromal haze and neovascularization; ↓IL-1β/IL-8/TNF-α, ↓M1 genes, ↑IL-10 and M2 markers	Suggests MSC-exo may promote healing while limiting inflammation/scarring	19
ASC-exo	In vitro + in vivo (cryoinjury model)	Improved CEnC migration/wound healing in vitro; improved endothelial recovery in vivo; mechanistic shifts consistent with reduced senescence/autophagy and altered pathways (lysine degradation, TGF-β, p53)	Potential cell-free strategy for corneal endothelial disorders given limited native endothelial regeneration	20
MSC-CM exosomes (exosome-dependent effect)	In vitro + in vivo (mouse 2 mm epithelial wound) + storage testing	MSC-CM promoted dose-dependent proliferation/migration and faster closure; effects diminished with exosome-depleted CM; 72 hours CM >48 hours CM; bioactivity preserved up to 4 weeks at 4°C	Positions exosomes as key active component of MSC-CM; supports exosome-based dosing standardization and practical storage for translation	21
Mesenchymal stromal cell–derived exosomes	In vitro + in vivo (mouse 2 mm epithelial debridement)	Faster wound closure in vitro; significantly greater healing at 24 hours in vivo (77.5% ± 3% vs. 41.6% ± 7%; *P* < 0.05)	Supports stromal/MSC-exos as a candidate treatment for corneal epithelial wounds	15
SEs	In vivo (mouse epithelial debridement) + in vitro (hCEC/hLEC assays)	Accelerated closure in vivo; ↑integrin α6/β4, TGF‑β1, TSP1 (TGF‑β1 up early then down by 72 hours); enhanced proliferation, migration, and mitochondrial function in vitro	Identifies saliva as a novel, noninvasive exosome source with potential for corneal wound-healing therapy	22
iPSC-derived exosomes vs. MSC-derived exosomes	In vitro (hCECs) + in vivo (rat epithelial defect model); direct comparative study	Both accelerated wound healing; iPSC-exo showed superior healing. In vitro, iPSC-exo increased hCEC proliferation and migration and reduced apoptosis more than MSC-exo. Both upregulated cell-cycle regulators (cyclin A, cyclin-dependent kinase 2), with stronger effects from iPSC-exo	Suggests iPSC-exo may be more potent than MSC-exo for corneal epithelial repair	23

GSK-3β, glycogen synthase kinase 3β; hCEnC, human corneal endothelial cell; irrPTK, irregular phototherapeutic keratectomy.

**Table 2.** Peptides and Key Outcomes

Peptide	Injury	Level of Evidence	Key Outcomes	Clinical Relevance	Reference
Histatin-1	Corneal epithelial wound (in vitro)	In vitro	Faster epithelial wound closure; increased cell spreading/pathfinding; minimal toxicity	Candidate topical prohealing peptide for epithelial defects	31
Histatin-1	An 8-mm corneal epithelial defect (rabbit)	In vivo (rabbit; dose-ranging)	Faster recovery across doses; higher % healed area/hourly healing; no adverse effects	Supports in vivo efficacy/safety for corneal epithelial injury	32
Histatin-5 (and SHRGY motif)	Corneal epithelial wound (in vitro) + 2-mm epithelial defect model (in vivo)	In vitro + in vivo	Promoted migration/spreading and faster closure; SHRGY residues required; SHRGY alone matched histatin-5 efficacy	Identifies a minimal active motif for streamlined peptide development	34
CGRP	A 2-mm epithelial injury model	In vitro + in vivo	Accelerated closure; reduced opacity/thickness/scarring/endothelial loss; reduced TGF-β1–linked fibrosis and inflammatory cell responses	Regenerative, antifibrotic, anti-inflammatory candidate for corneal injury	36
Substance P + IGF-1	Photorefractive surface ablation (rabbit)	In vivo (rabbit)	Accelerated epithelial wound healing; consistent with enhanced neurokinin-1 receptor–mediated epithelial migration	Potential adjunct to speed epithelial recovery after surface ablation	41
Decorin	Corneal abrasion model	In vivo	Accelerated epithelial healing; promoted sensory nerve regeneration; decreased neutrophil recruitment; altered TGF-β/CSPG4 expression	Dual epithelial + nerve repair approach for corneal injury	44
NGF vs. KGF-2	Corneal alkali burn (mouse)	In vivo (mouse)	Both improved early re-epithelialization and reduced inflammation/opacity/neovascularization; NGF stronger antineovascularization; ↓MMP-2/9 and TGF-β with NGF	NGF highlighted as an adjuvant option for chemical burns	62
VIP	Corneal alkali burn (rabbit)	In vivo (rabbit) + mechanistic follow-up	Improved ulceration/epithelialization (not significant); reduced polymorphonuclear leukocyte levels; sonic hedgehog-dependent healing mechanism suggested	Possible anti-inflammatory adjunct; efficacy needs stronger confirmation	63,64
SST	Corneal alkali injury (mouse)	In vivo (mouse) + in vitro/mechanistic	Significantly faster closure in vivo; no direct hCEC proliferation/migration boost in vitro; ↑vascular endothelial growth factor; immunomodulation (↓TNF-α/nuclear factor-κB; M1→M2 via SSTR5)	Supports microenvironment/immunomodulatory peptide strategy for chemical injuries	67–69

#### Amnion

AM is an agent derived from the placenta that has been shown to exhibit many regenerative and healing properties through anti-inflammatory, antiangiogenic, antifibrotic, proepithelialization, and antimicrobial/antiviral mechanisms. Its use has expanded to many fields of ophthalmology, including corneal reconstruction, conjunctival repair, and strabismus surgery.^9^

One study explored the effects of AM and UC (AMUC) eye drops on the healing of corneal abrasions in murine models. In AMUC-treated mice, corneal epithelization was faster, indicated by the smaller epithelial defect at 12 hours (*P* = 0.002), 1 day (*P* = 0.016), and 2 days (*P* = 0.04), and the incidence of complete corneal smoothness was higher, as well. Complete epithelialization was quicker in the AMUC group (3.15 ± 1.44 vs. 4.00 ± 1.63 days; *P* = 0.06), however, this difference was not statistically significant. Mechanistically, CD45^+^ cells (inflammatory response) infiltration was significantly decreased in the peripheral cornea and Ki-67^+^ cells (proliferation response) were promoted in the central cornea for the AMUC group, showing anti-inflammatory and cell proliferation properties. This study highlights AMUC as a noninvasive, convenient approach for the regenerative repair of corneal abrasions and warrants more clinical trials.^10^

#### Exosomes

Exosomes are extracellular vesicles (EVs) (∼40–150 nm) that play a vital role in cell-to-cell communication.^11^^,^^12^ They are membrane bound and carry bioactive cargo, including proteins, nucleic acids, and metabolites, thereby influencing cellular signaling and function. Because of their small size and biocompatibility, major biological membranes can be crossed.^13^ Studies have shown that various corneal cells release exosomes, showing a potential role for exosomes as a therapeutic for corneal injury.^14^^–^^17^

One study compared exosomes from human corneal epithelial cells (hCECs), human corneal fibroblasts (hCFs), and human corneal endothelial cells and their effects on scratch-wounded hCECs. hCECs showed the most noticeable uptake (vs. hCF exosomes), whereas hCF-derived exosomes produced the greatest increase in proliferation. Overall, all exosome treatments accelerated wound closure compared with controls, with hCEC-derived exosomes driving the fastest closure. Genetically, the different exosomes affected signal transduction mediators differently. HSP27 (cell proliferation and adhesion mediator) presence was increased by hCEC exosomes. Moreover, the phosphorylation of glycogen synthase kinase 3β, p38α (mitogen-active protein kinase [MAPK]), and β-catenin in hCECs was increased by hCEC and hCF exosomes, but eliminated by human corneal endothelial cell exosomes. This study shows the healing properties of corneal-derived exosomes and reveals their different cell-type–specific, gene-altering interactions.^18^

Another study examined mesenchymal stem cell exosomes (MSC-exo) and found MSC-exo treatment induced faster wound closure and more rapid cell proliferation and migration using an in vitro scratch assay. A rat irregular phototherapeutic keratectomy model was treated with MSC-exo and confirmed the in vitro results showing greater wound closure on day 5. Notably, the corneas treated with MSC-exo had no stromal haze and suppressed neovascularization. Mechanistically, the levels of proinflammatory cytokines interleukin (IL)-1β, IL-8, and tumor necrosis factor (TNF)-α were decreased and M1 macrophage-associated genes were downregulated, whereas anti-inflammatory IL-10 levels increased and M2 markers were upregulated, suggesting an immunomodulatory basis for the therapeutic effect of MSC-exo.^19^

One study evaluated adipose MSC-derived exosomes (ASC-exos) as a therapy to promote corneal endothelial cell regeneration. ASC-exos enhanced CEnC migration and wound healing in vitro and improved endothelial recovery in a cryoinjury-based in vivo model. Mechanistically, ASC-exos were observed to induce shifts in the cell cycles by inhibiting senescence and autophagy pathways such as lysine degradation, the transforming growth factor (TGF)-β signaling pathway and the p53 signaling pathway.^20^ Because corneal endothelial cells are unable to regenerate, ASC-exos represent a potential cell-free therapeutic strategy for corneal endothelial diseases.

Another study examined whether MSC-derived exosomes drive the corneal wound-healing benefits of bone marrow MSC-conditioned media, which is used to capture MSC-secreted factors while avoiding the potential adverse effects of cell therapy. In vitro and in vivo (a 2-mm epithelial wound in a corneal mouse model) results show promoted dose-dependent cell proliferation, migration, and faster wound closure. These healing effects were diminished in the exosome-depleted MSC-CM, supporting exosomes as key active components. MSC-CMs conditioned 72 hours exhibited greater wound healing effects than MSC-CMs conditioned for 48 hours. The study also examined the effects of varying storage conditions, finding that there is no loss of biofunction at up to 4 weeks of storage at 4°C when compared with MSC-CM that was not frozen. This study elucidates the important role of MSC-exos as active ingredients for cell proliferation and migration promoted by MSC-CMs; the dose dependence on MSC-exos sheds light on standardization of dosing for MSC-CM based on the concentration of exosomes.^21^

Another study examined mesenchymal stromal cell-derived exosome effects on corneal epithelial wound healing. In vitro, the exosome group caused faster wound closure and thus greater cell proliferation. In vivo, a 2-mm corneal debridement was performed on murine models. At 24 hours, the exosome-treated corneas showed a significant decrease in wound size compared with the control (77.5% ± 3% healed vs. 41.6% ± 7%; *P* < 0.05). These results show promising potential for mesenchymal stromal cell-derived exosomes as an endogenous therapeutic agent for corneal wounds.^15^

A recent study explored the healing effects of salivary exosomes (SEs). SEs carry many healing-promoting factors, such as antimicrobial peptides, growth factors, enzymes, and cytokines, making it a potentially effective candidate for corneal wound healing.^22^ In vivo, epithelial debridement was performed on mice and treated with SEs. The SE-treated corneas showed significantly accelerated wound closure, as well as an upregulation of integrin α6, integrin β4, TGF-β1, and TSP1 levels. Interestingly, the levels of TGF-β1 levels were found to be elevated in the first 24 hours and decreased by 72 hours, possibly leading to an enhanced initial healing response followed by a tempered healing response. In vitro, hCEC and human limbal epithelial cell (hLEC) proliferation, migration, and mitochondrial function were found to be enhanced. These results show SE as a novel exosome source with potential for corneal wound healing.^22^

A direct comparative study tested induced pluripotent stem cell (iPSC) exosomes against MSC exosomes in rat epithelial defect models. Both exosome types showed accelerated wound healing, but iPSC exosomes showed superior effects. In vitro, hCEC proliferation, migration, and inhibition of apoptosis was greater in the iPSC group. Mechanistically, both exosomes were found to upregulate cell cycle regulators including cyclin A and cyclin-dependent kinase 2, promoting cell cycle progression, although iPSC exosomes showed stronger effects.^23^ The enhanced effects in iPSC exosomes may be attributed to richer proteomic content in comparison with MSC-exos.^24^

Overall, there are a plethora of different sources of exosomes that carry great therapeutic potential for the acceleration of corneal healing properties (Table 1). Exosomes represent a cell-free therapy that carries the benefits of stem cells without the looming uncertainties. Stem cell–based therapy carries ethical and clinical issues, including tumorigenicity, teratocarcinoma, and uncertain fate possibility, as well as isolation and storage issues.^25^ However, exosomes carry no tumor potential, are easier to isolate, circulate through the body easier (owing to their small size), and show no long-term toxicity, illustrating exosomes as an alternative to cell-based therapies. It has been found recently that stem cells derive their regenerative abilities from paracrine secretion of active molecules rather than direct regeneration and differentiation. Exosomes have been found to induce similar effects on wound healing pathways, supporting its use as an alternative to stem cells with comparable outcomes.^11^^,^^25^ Exosomes remain a novel treatment and warrants further studies including comparison studies among the types of exosomes.

#### Stem Cells

One study examined the healing effects and mechanism of corneal stromal stem cell (CSSC)-EVs on corneal debridement wounds via corneal mouse models. Both CSSCs and CSSC-EVs were found to suppress visible scarring, suppress expression of fibrotic genes in wounded corneas, suppress neutrophil infiltration, and suppress expression of inflammatory messenger RNA 3 days after wounding, showing that CSSC-EVs replicated the regenerative capabilities of CSSCs. Moreover, the Alix protein was identified to be required for the microRNA (miRNA) transfer of CSSC-EVs into exosomal EVs. When the Alix protein was knocked out of the CSSC-EVs, corneal regeneration did not occur, indicating the importance of miRNA in this process. Corneal wounds treated with these EVs developed scars and expressed the inflammatory cell infiltration-associated genes. This study highlights the important role miRNA plays in wound regeneration.^26^

#### Platelet-rich Plasma

PRP is an autologous blood derivative with increased concentrations of platelets rich in growth factors. These growth factors (e.g., TGF-β, epidermal growth factor [EGF], vascular endothelial growth factor, platelet-derived growth factors, fibroblast growth factor, and insulin-like growth factor [IGF-1]) signal and promote proliferative, immunomodulatory, and angiogenic mechanisms.^27^ PRP therapy has expanded into fields beyond ophthalmology and is especially efficacious in orthopedic indications.^28^

One study evaluated the healing effects of PRP eye drops and the maintenance of its sterility during storage. Owing to the lack of chemical preservatives, sterility of PRP and autologous serum (AS) eyedrops remain a prevalent issue. This study examined this sterility and found that both PRP and AS drops maintained sterility when stored for 4 weeks at 4°C. In vivo, 2-mm (diameter) epithelial wounds were performed on mouse models. Both in vitro and in vivo results show PRP stored for 4 weeks at 4°C promoted the greatest corneal wound healing compared with AS eye drops (0 and 4 weeks stored) and PRP (0 weeks stored). Important growth factors—EGF and TGF-β1—levels were found to be increased during storage, which may be attributed to the enhanced healing properties of the 4-week stored PRP. However, fibronectin expression did not show any changes before and after storage. PRP remains a relatively new therapy and continues to lack standard medical protocol.^27^ This study shows PRP as a therapeutic for corneal disorders that have unmet medical needs.^29^

#### Peptides

Recently, histatins have been explored for their wound healing effects. Histatins are a family of antimicrobial peptides that exhibit numerous therapeutic effects.^30^ One study explored histatin-1 on corneal epithelial wound healing. In vitro, histatin-1 was found to enhance corneal epithelial wound healing with minimal toxicity and appeared to enhance spreading and pathfinding, showing the potential healing effects of histatin-1 in vitro.^31^

A following study probed these findings further with an in vivo model using 8-mm epithelial wounds in rabbits. The wounds were treated with varying concentrations of histatin-1. They found that all histatin-1 treated groups wounds had faster recovery, greater percent recovered area, faster hourly healing rates, and no adverse effects were observed. This was the first in vivo study evaluating histatin-1 and its efficacy in corneal wound healing, showing a potential role of histatin-1 in corneal injury treatment.^32^ Further studies showed histatin-1 is an endogenous ligand of the sigma-2 receptor (also known as TMEM97), which is a receptor involved in cell proliferation, differentiation, and apoptosis shedding light on histatin-1’s mechanism.^30^^,^^33^

**Figure 2.**
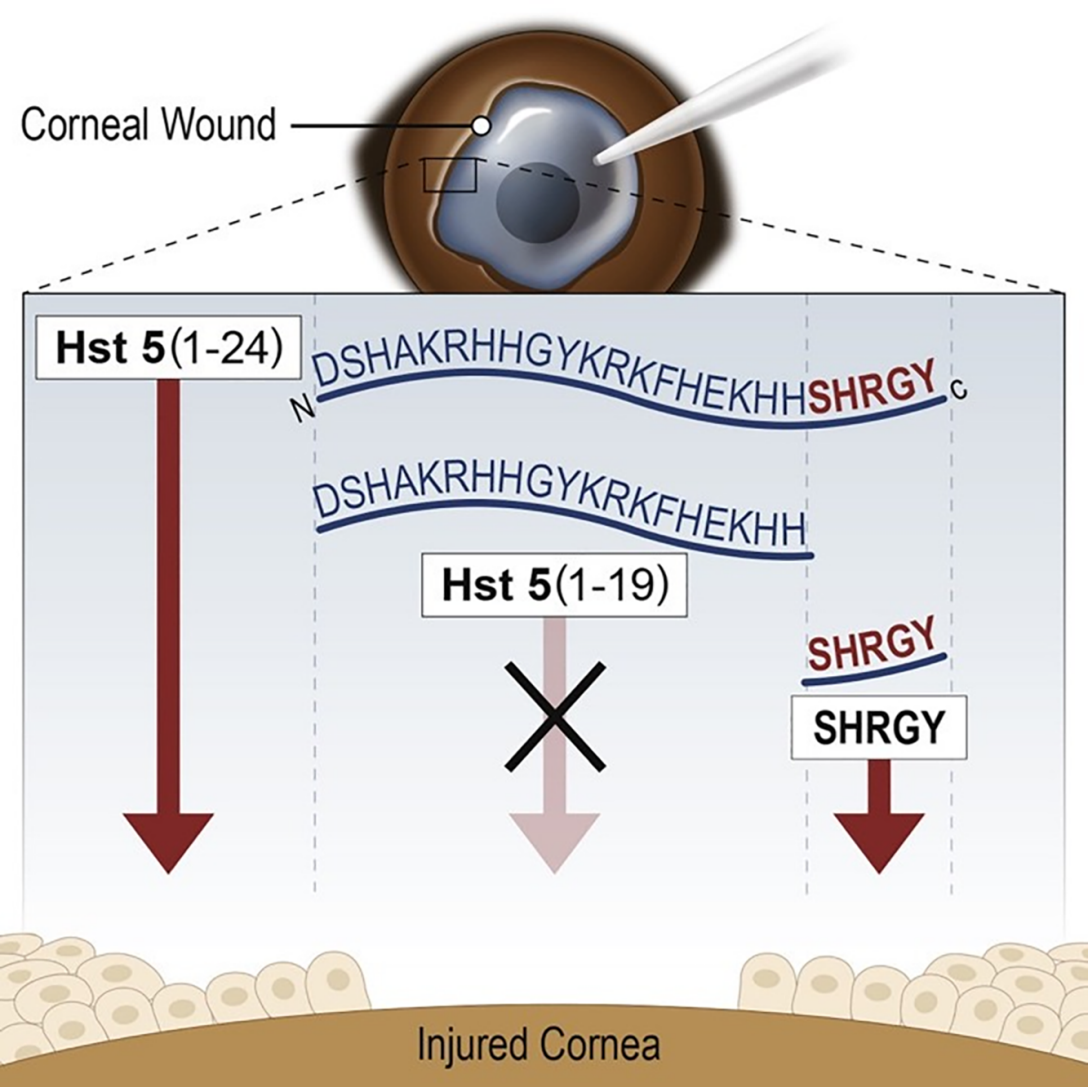
Schematic of histatin 5 function and absence for the injured cornea and wound healing, as well as the effects of SHRGY-containing variants of histatin 5 on corneal wound healing. (Figure reprinted from Shah D et al.^34^ Wound Healing Properties of Histatin-5 and Identification of a Functional Domain Required for Histatin-5-Induced Cell Migration. Mol Ther Methods Clin Dev. 2020 Mar 31;17:709–716 with permission under Creative Commons License.)

**Figure 3.**
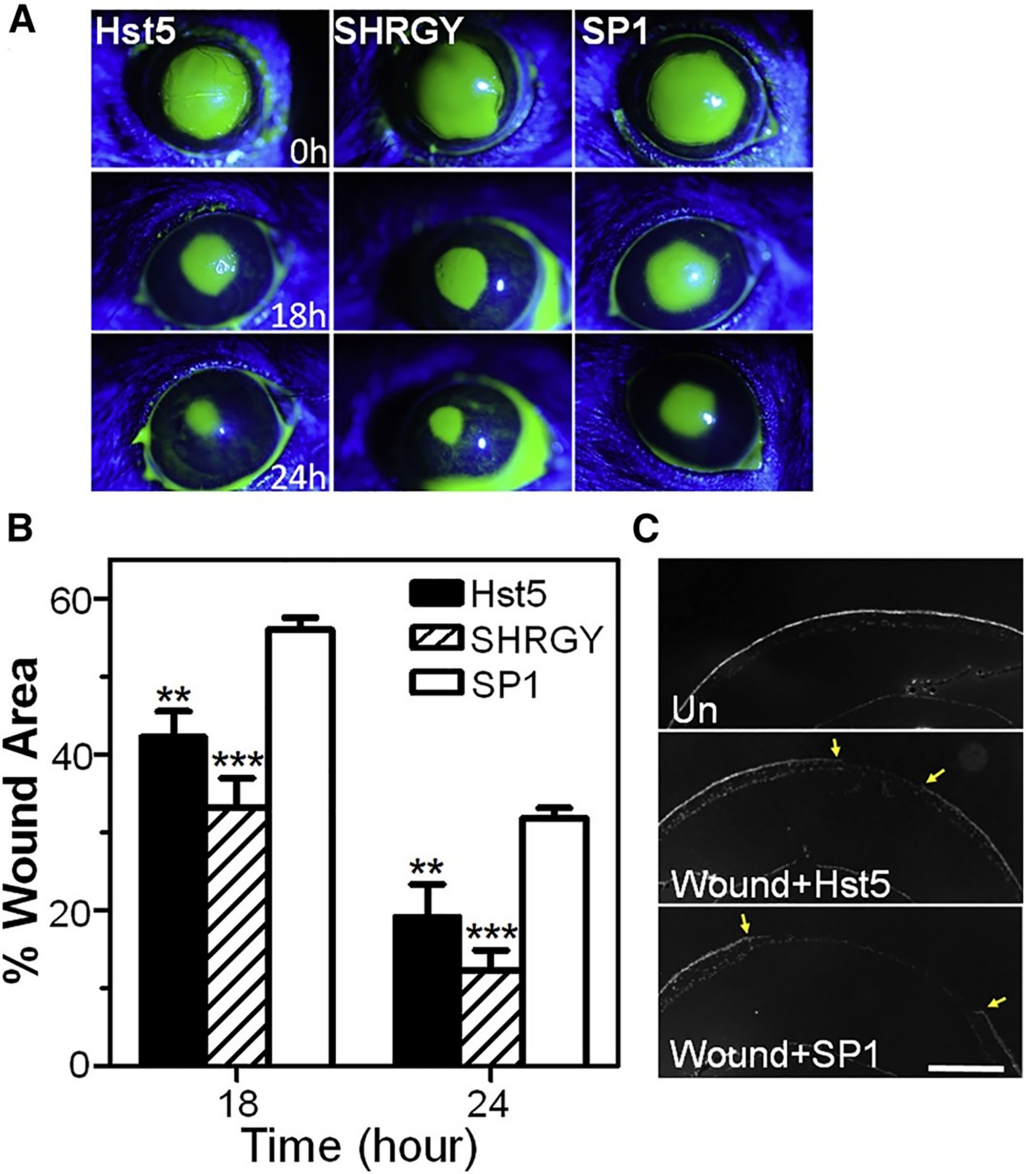
Corneal epithelial wound injury on fluorescein stain at time points of initial injury (0h), 8 hours (8h) and 24 hours (24h) (**A**) with histatin 5 (Hst5), C-terminal SHRGY residues, and scrambled peptide number 1 (SP1), which does not contain SHRGY. (**B**) Wound area of the course of 18 hours and 24 hours among Hst5, SHRGY, and SP1. (**C**) Comparison of a corneal wound with Hst5 against a wound with SP1. (Figure reprinted from Shah D et al.^34^ Wound Healing Properties of Histatin-5 and Identification of a Functional Domain Required for Histatin-5-Induced Cell Migration. Mol Ther Methods Clin Dev. 2020 Mar 31;17:709–716 with permission under Creative Commons License.)

Another study explored histatin-5 and its wound healing properties. In vitro*,* histatin-5 was found to promote wound healing, epithelial migration, and cell spreading. In serial truncations, the authors found that the C-terminal SHRGY residues were critical to promote migration. In vivo, mice with 2-mm epithelial wounds were treated with topical histatin-5 and SHRGY—an isolated pentapeptide from histatin-5—finding increased rates of wound healing in the murine model (Fig. 2). Notably, the SHRGY domain alone induced the same healing as histatin-5. The authors also compared this SHRGY with scrambled peptide number 1, which does not contain SHRGY, which is thought to be critical for promoting migration (Fig. 3^3^). This study confirmed the previous results of histatin studies, identified a minimal peptide motif for potential development, and warrants more studies on the efficacy and safety of histatin-5 and histatins, in general.^34^

Calcitonin gene-related peptide (CGRP) is a neuropeptide expressed by the corneal nerve. CGRP binds to its receptor which is a G-protein coupled receptor, mediating proinflammatory and anti-inflammatory activities. In addition to its sensory roles, CGRP has been shown to play an important role in corneal wound healing.^35^ One study examined its therapeutic use for corneal injury. In vivo, 2-mm epithelial injuries were topically treated with CGRP. The results showed that CGRP-treated groups had accelerated epithelial wound closure, reduced corneal opacity, and decreased corneal thickness, scar formation, and endothelial loss. Mechanistically, CGRP was found to reduce TGF-β1–mediated stromal fibroblast activation and tissue fibrosis, as well as reduce inflammatory responses, including neutrophil infiltration, macrophage maturation, and inflammatory cytokine production. These findings suggest CGRP as a regenerative, antifibrotic, and anti-inflammatory agent and should be further studied.^36^

Substance-P (SP) is a neuropeptide in the tachykinin family that plays a large role in cornea homeostasis and has been found to both enhance wound healing and amplify inflammatory responses.^37^^,^^38^ Previous studies have shown SP in combination with IGF-1 to promote corneal epithelial cell migration through neurokinin-1 receptor, protein kinase C, and the p38 MAPK activation pathway.^39^^,^^40^ A recent study assessed SP plus IGF-1 treatment in photorefractive surface ablations in rabbits, finding accelerated epithelial wound healing. SP plus IGF-1 is thought to increase corneal epithelial migration mediated by neurokinin-1 receptor.^41^^,^^42^

One study explored the proteoglycan, decorin, which has been previously found to exhibit nerve-regenerating properties in corneal sensory nerves.^43^ Corneal abrasion injury was induced and treated topically with decorin. Results show that sensory nerve regeneration in the central cornea was promoted, and epithelial wound healing was accelerated after 5 days of topical treatment. TGF-β and CSPG4 messenger RNA showed altered expression in decorin-treated corneas, and neutrophil recruitment was decreased. Dendritic cell levels were measured, and the decorin-treated group were found to have greater dendritic cell density than the saline-treated groups in the acute phase of corneal injury showing a correlation between the rapid epithelialization at 12 hours and dendritic cell density. Decorin is a novel agent with unique nerve-regenerating properties, representing a promising approach for future treatment.^44^

### Acute Chemical Burns

An acute chemical burn is a severe ocular injury with a relatively high incidence. One study found 15,865 cases of ocular chemical burn per year in the United States.^45^^,^^46^ Over a 4-year period, 144,149 chemical ocular burns were diagnosed across the United States and 1- and 2-year-olds represented the age group with the highest risk.^45^^,^^47^ Acute chemical burns can cause irreversible damage to the ocular surface and limbus.^48^ Chemical burns can occur from alkaline substances or acidic substances. Alkali burns involve the disruption of the cell membrane through lysis, allowing the alkaline substance to penetrate the cornea and anterior chamber, destroying tissue on its path.^47^^,^^49^^–^^51^ Conversely, acidic agents tend to cause less severe burns, because coagulation necrosis of denatured proteins can form a barrier that prevents further penetration.^47^^,^^52^^,^^53^ Corneal scarring and delayed healing result from corneal neovascularization and fibrosis, which are caused by excessive inflammatory factors. Thus, it is important that treatments target this physiological response, specifically, neutralizing inflammatory mediators, blocking immune cell recruitment, minimizing the extent of tissue damage, and promoting tissue regeneration.^54^

**Figure 4.**
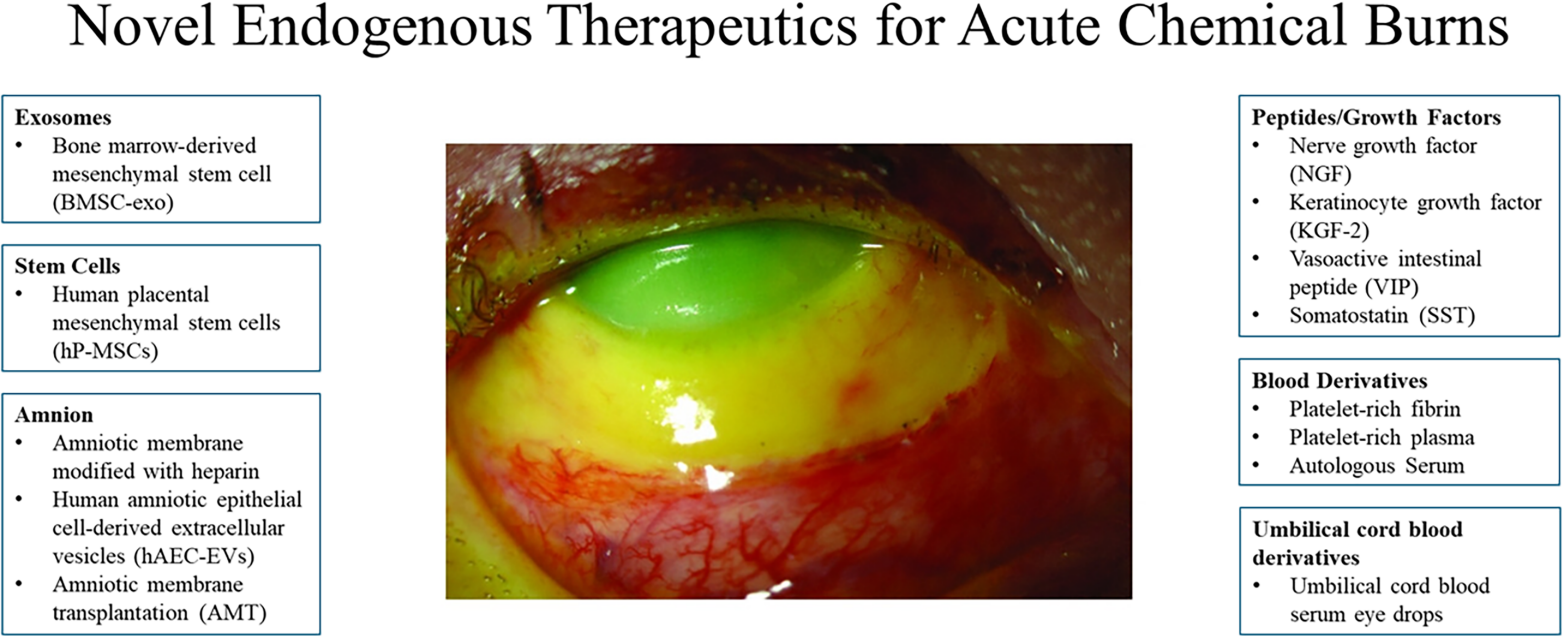
Schematic of novel therapeutics for acute chemical burns. Grade IV ocular burn. (Central image adapted from Eslani et al.^55^ The ocular surface chemical burns. *J Ophthalmol*. 2014;2014:196827.with permission under Creative Commons License.)

Managing chemical burns involves the removal of the chemical agent, irrigation for neutralization, promotion of epithelialization, minimization of ulceration, and inflammation control.^48^ Ascorbate and tetracyclines are used to promote tissue repair and minimize ulceration. Inflammation control is achieved through corticosteroids, progestational steroids, nonsteroidal anti-inflammatory drugs, and citrate. Prophylactic topical antibiotics are used for preventing infection. In severe cases, surgical management can be warranted.^48^ However, the current treatment for ocular burns can be limited, including the adverse effects of steroids and low availability of donor corneas for transplantation.^54^ Thus, more comprehensive and accessible treatment is warranted. This section reviews endogenously sourced and allogenically sourced therapeutic agents—amnion, stem cells, growth factors, PRP, peptides, and UC serum (UCS)—as novel treatments for acute chemical burns. Figure 4 shows a summary of these treatments.

#### Amnion

Zhao et al.^56^ modified AM with heparin. Performing various in vitro tests, they found that the structure, material properties, and biocompatibility did not differ from unmodified AM. Moreover, heparin-modified AM (AM-HEP) had improved loading capacity and sustained release of epithelial growth factor as well as quicker absorption. Cell proliferation and cell migration were shown to be promoted, where AM-HEP combined with EGF (AM-HEP@EGF) promoted the fastest healing in corneal epithelial cell cocultures. In vivo, AM-HEP@EGF–corneal alkali burns in mice models had higher transparency, faster wound healing rates, greater downregulation of inflammatory-related genes (i.e., IL-1β, IL-6, and TNF-α), and greater epithelialization. AM-HEP@EGF is a high-potential candidate for therapeutic use in corneal alkali burns and could be used for many more biological functions as well.^56^

Hu et al.^57^ studied human amniotic epithelial cell-derived EVs (hAEC-EVs) and their potential therapeutic effects. Using corneal rabbit models, hAEC-EVs were applied via eye drops and injections and compared with a phosphate-buffered saline control group. Hu et al.^57^ found that hAEC-EVs induced rapid re-epithelialization and formed a thinner, more orderly stromal structure, resulting in less scar formation and greater corneal transparency. In vitro, they found increased cell proliferation and migration in both hCECs and human corneal stromal cells, demonstrating the role of the focal adhesion signaling pathway in this process. Hu et al.’s experiments^57^ show that hAEC-EVs are great potential candidates for corneal alkali burn treatment.

A randomized controlled trial (RCT) compared the efficacy of AM transplantation (AMT) and UCS drops in grade III, IV, and V (Dua's classification) acute chemical burns. This RCT divided the patient population into three groups (15 patients each): conventional treatment, AMT with conventional treatment, and UCS with conventional treatment. The results showed that AMT and UCS groups achieved complete epithelialization faster than conventional treatment groups (56.7 ± 14.9 days, 22.0 ± 10.2 days, and 22.9 ± 10.1 days in the conventional treatment, AMT, and UCS groups, respectively). Pain scores and corneal clarity were also assessed, showing greater improvement in the UCS group.^58^

In contrast, a more recent RCT strictly evaluated the use of AMT combined with conventional treatment in patients with Roper–Hall grade IV ocular chemical injuries, comparing it with conventional treatment alone (lubricating gel and eye drops, chloramphenicol, betamethasone, homatropine, oral vitamin c, and doxycycline). Interestingly, the results showed no differences in corneal epithelial defect healing time (72.6 ± 30.4 days [range, 21–180 days] vs. 75.8 ± 29.8 days [range, 46–170 days]; *P* = 0.610) or mean BCVA (2.06 ± 0.67 logarithm of the minimum angle of resolution [logMAR] [range, 0.4–2.6 logMAR] vs. 2.06 ± 0.57 logMAR [range, 1.0–2.9 logMAR]; *P* = 0.85). AMT-treated burns showed less central neovascularization, but this difference was not statistically significant (22 eyes [73.3%] vs. 16 eyes [53.3%]; *P* = 0.108). Thus, this RCT showed no therapeutic advantage with AMT-combined treatment in severe chemical injuries.^59^

The evidence surrounding AMT for the treatment of acute chemical burns remains in question. Although some RCTs point to efficacious outcomes, other studies (including RCTs) show otherwise. It may be of benefit to modify the AM as Zhao et al.^56^ did with heparin.

#### Exosomes

One study explored exosomes isolated from bone marrow-derived MSCs (BMSC-exos). BMSC-exos were tested in vitro through cocultures with hCECs, and they found that both hCEC proliferation and migration were promoted. Moreover, increasing the concentration of BMSC-exos increased the amount of proliferation and migration. The study also found that the mechanism of BMSC-exos involved the p44/42 MAPK pathway. In vivo, the BMSC-exos were injected into alkali-burned mice corneal tissues and found superior relief regarding the inflammation, fibrosis, and vascularization of the corneal tissue, including lower levels of α-smooth muscle actin (a fibrosis marker) and CD31 (a vascularization marker). BMSC-exos may be a promising therapeutic agent regulated via the p44/42 MAPK pathway.^60^

#### Stem Cells

Another study probed human placental MSCs and their subconjunctival administration targeting inflammatory cytokines. In vivo, MSC-treated alkali burns showed superior re-epithelialization and corneal opacity, including rapid amelioration of corneal opacity in MSC-treated group within 14 days. Histological analysis showed suppression of inflammatory reaction with reduced TNF-α expression and significant suppression of IL-1β, monocyte chemoattractant protein-1, and matrix metalloproteinase-9 expression. Antiapoptotic effects were also observed as well. human placental MSCs show therapeutic efficacy similar to that of BMSCs, with easier harvesting abilities and greater growth abilities.^61^

#### Peptides and Growth Factors

Growth factors play key roles in the migration and proliferation of damaged tissue. Nerve growth factor (NGF) and keratinocyte growth factor-2 (KGF-2) are released in response to corneal injuries and regulate healing.^62^ A growth factor study evaluated the effects of NGF and KGF-2. Previously, the therapeutic effects of NGF had not been studied in corneal alkali burns. The study used corneal alkali burn mouse models and found that both topical application of NGF and KGF-2 were effective in early re-epithelization and reducing inflammation, corneal opacity, and neovascularization when compared with the control group. Compared with KGF-2, NGF inhibited neovascularization superiorly. MMP-2, MMP-9 and TGF-β levels were decreased in the NGF group, showing positive immunoreactivity response. This study particularly highlights NGF as an effective adjuvant therapy for corneal alkali burns.^62^

Vasoactive intestinal peptide (VIP) is a neuropeptide in the secretin/glucagon superfamily and is known to be an inhibitory neurotransmitter in the central and peripheral nervous systems.^35^ One study assessed the local application of VIP on alkali-burned rabbit models, finding positive effects on corneal ulceration and corneal epithelialization although the differences failed to reach statistical significance. The study also found decreased levels of polymorphonuclear leukocytes in VIP groups. Polymorphonuclear leukocytes are thought to be major players in the events after an alkali burn.^63^ A recent study found that VIP's healing effects occur in a sonic hedgehog-dependent manner.^64^

Somatostatin (SST) is a cyclic peptide in the SST family, most notable for its neuroendocrine effects.^35^ SST has been detected in tear fluid, and the cornea has been shown to express SST receptors.^35^^,^^65^^,^^66^ In alkali-induced corneal injury mouse models, topical application of SST was found to significantly accelerate wound closure. Interestingly, in vitro results showed that hCEC proliferation and migration were not directly enhanced and failed to activate ERK1/2 and p38 signaling pathways. Instead, vascular endothelial growth factor protein expression was upregulated, suggesting its healing mechanism through the modulation of the microenvironment and growth factors present rather than direct stimulation of epithelial cells.^67^

Recent work has shown the mechanism to be immunomodulatory. SST suppressed TNF-α and nuclear factor-κB inflammatory pathways and shifted macrophage polarization from proinflammatory M1 phenotype to the reparative M2 phenotype. The SST receptor type 5 pathway was also shown to be a key mediator.^68^^,^^69^ Table 2 summarizes these peptides and their key outcomes.

#### Blood Derivatives

Platelet-rich fibrin (PRF) represents the second generation of blood-derived platelet concentrate. PRF differs mainly from PRP in its simpler preparation. PRF is completely autologous and prepared without any anticoagulants, bypassing the inhibition of wound healing cascades seen in PRP. Despite the simpler preparation, PRF remains rich in growth factors, leukocytes, and cytokines.^70^

One study treated three corneal burn patients with PRP eye drops and/or a PRF graft. The first case (a chemical ocular burn from cement powder) was initially treated with antibiotics and AS for 1 week with no results, but after PRP treatment achieved complete function and morphological recovery. The second case (a chemical ocular burn from paint thinner) was similarly treated for 1 week without results, but achieved complete recovery after PRP treatment. The third case (a chemical ocular burn from quicklime) was treated for 8 weeks without results. After treatment with a PRF membrane graft on days 0, 5, and 7, corneal healing reached about 95%. Although this study was limited its sample size, PRP/PRF treatment showed remarkable results and larger studies should be conducted to confirm its efficacy.^71^

#### Umbilical Cord Blood Serum

UCBS is a derivative of UC blood. UCBS is a reservoir for growth factors, neurotrophic factors, and cytokines with wound-healing and anti-inflammatory properties. UCBS has seen use for the treatment of various ocular surface disorders including dry eye, persistent epithelial defects, and neurotrophic keratitis.^72^^,^^73^ Previous studies have shown UCBS to exhibit greater efficacy than AS or artificial tears.^74^^,^^75^

A more recent RCT compared the efficacy of UCBS and AS in ocular surface disorders including acute chemical burn. Twenty-one eyes afflicted with acute chemical burn were evaluated and, using Dua's classification, 11 eyes had grade III, 6 had grade IV, and 4 had grade V chemical injury. UCBS outperformed AS in the treatment of these burns, indicated by faster re-epithelialization, improvement in eye sensation score, improvement in epithelial defect diameter, and a greater decrease in limbal ischemia. This RCT found superior ocular surface restoration properties in UCBS compared with AS, confirming previous results and reaffirming comparative data for future acute chemical burn therapy.^76^

### Chronic Corneal Disease

Although this review focuses primarily on acute corneal injuries, the therapeutics discussed above exhibit can extend to chronic corneal disease, including neurotrophic keratopathy (NK) and diabetic keratopathy (DK) where corneal wound healing is chronically disrupted. Here, we briefly discuss the endogenous therapeutics used in chronic corneal disease.

NK is a degenerative condition characterized by decreased innervation of the cornea that can result in persistent epithelial defects, ulceration, stromal melting, and perforation. Notably, corneal innervation is critical for the provision of trophic factors like SP and CGRP to the epithelium.^77^ For stage 2 NK treatment, promotion of epithelial healing and prevention of corneal degeneration are the goals, similar to the treatment goals of acute corneal injuries, lending endogenous therapeutics as great candidates for this treatment.^77^ AS tears have seen efficacy for NK and its combination with SP plus IGF-1 showed upregulation of corneal growth and migration.^77^^–^^79^ UCBS and peripheral blood have shown efficacy resulting in resolution of epithelial defects.^77^^,^^80^^–^^82^ The advanced form of NK, stage 3, has been tested and treated with a recombinant form of NGF (cenergermin), increasing corneal healing rates.^77^^,^^83^^,^^84^ Surgically, AMT has shown greater efficacy than conventional approaches although its efficacy has been found to be similar to AS.^77^^,^^85^^,^^86^ More novelly, plasma rich in growth factors has shown positive effects in treatment of NK.^77^^,^^87^^–^^89^ NK can be a debilitating disease and the use of endogenous agents such as the neuropeptides involved in the pathophysiology should be the subject of further study.

DK is a degenerative corneal disease in patients suffering from diabetes mellitus. Clinical manifestations of DK include corneal ulcers, delayed re-epithelialization, reduced corneal sensitivity, and corneal edema. The corneal wound healing mechanism is thought to be disrupted in DK, leading to epithelial defects and corneal erosion.^90^ The synergistic effects of SP and IGF-1 have been well-documented in DK, showing improvements in corneal resurfacing.^90^ EGF, PRP, and plasma rich in growth factor have also been used and effective in DK patients, improving corneal wound healing.^90^^–^^94^ Stem cell research for corneal diabetes has targeted limbal epithelial stem cells, MSCs, embryonic stem cells, and iPSCs.^90^^,^^95^^–^^99^ Recently, exosomes derived from MSCs and BMSCs have shown restorative effects.^100^^,^^101^

Overall, NK and DK are chronic corneal disorders that exhibit corneal degeneration, making corneal endogenous biologics promising strategies for restoring epithelial integrity and prevent further damage. Further studies should further define the disease mechanisms and clinical use of these therapeutics in chronic keratopathies.

## Translational Considerations

It should be noted that many of these therapies are in early stages of development and testing and that there are translational barriers to bring these therapies to widespread clinical use. Many of the therapies listed, such as iPSC and histatin, have been used in animal studies as well as in human epithelial cell cultures. However, these models differ from real-life clinical application for the human corneal epithelium when facing an acute corneal injuries such as ongoing exposure (e.g., chemical injury or foreign body in the fornixes). Other challenges or considerations include sourcing materials and resources and whether these therapies outweigh the current standard of care. The practicality of sourcing and maintaining certain resources such as UC blood for acute corneal injuries may not be practical to implement globally. Regulatory pathways are also an area of discussion if these endogenously sourced agents come from local sources and must be accessed through national and regional regulations. Additionally, a pertinent question is that, even if certain endogenous sources increased the rate of closure of epithelium or other biometrics commonly used to evaluate these early stage endogenous therapies, would this impact the overall procedural outcome (e.g., decrease the rate of optical keratoplasty)? Although the preliminary data indicate that these would likely help to decrease the risk of requiring procedures to improve vision, these questions must be answered for translational growth. This work will require the initiation of phase 1 clinical trials to demonstrate safety and then subsequent phases for efficacy. Of the endogenous agents listed, all have certain pros and cons. Relatively easily sourced agents such as histatins, blood derivatives, and growth factors may be the most feasible for clinical translation. Additionally, agents that are easy to apply (e.g., eye drops) are another property that can lower the translational barrier. Ultimately, further research is most certainly necessary before clinical implementation for these early endogenous peptides.

## Conclusions

Endogenous therapeutic agents have been shown to be a promising therapeutic strategy in acute corneal injuries. However, future research is required before clinical adoption and implementation. Research including further in vivo studies and staged randomized control trials will be critical to understand the safety profiles and therapeutic efficacy in preserving vision after acute injury to the cornea. In conjunction with current standard practice, endogenous therapeutic agents may have a future role in preserving vision for patients.
